# Do health partnerships with organisations in lower income countries benefit the UK partner? A review of the literature

**DOI:** 10.1186/1744-8603-9-38

**Published:** 2013-08-30

**Authors:** Felicity AE Jones, Daniel PH Knights, Vita FE Sinclair, Paula Baraitser

**Affiliations:** 1 Medical School, King’s College London, London, UK; 2 School of Clinical Medicine, University of Cambridge, Cambridge, UK; 3 King’s Centre for Global Health, Kings College London and Kings Health Partners, London, UK

## Abstract

**Background:**

Health partnerships between institutions in the UK and Low or Lower- middle Income Countries are an increasingly important model of development, yet analysis of partnerships has focused on benefits and costs to the Low and Lower- Middle Income partner. We reviewed the evidence on benefits and costs of health partnerships to UK individuals, institutions & the NHS and sought to understand how volunteering within partnerships might impact on workforce development and service delivery.

**Methods:**

A systematic review of both published literature and grey literature was conducted. Content relating to costs or benefits to the UK at an individual, institutional or system level was extracted and analysed by thematic synthesis. The benefits of volunteering described were mapped to the key outcome indicators for five different UK professional development structures. A framework was developed to demonstrate the link between volunteer experience within partnerships and improved UK service delivery outcomes.

**Results:**

The literature review (including citation mapping) returned 9 published papers and 32 pieces of grey literature that met all inclusion criteria. 95% of sources cited benefits and 32% cited costs. Most literature does not meet high standards of formal academic rigor. Forty initial individual benefits codes were elicited. These were then grouped into 7 key domains: clinical skills; management skills; communication & teamwork; patient experience & dignity; policy; academic skills; and personal satisfaction & interest. A high degree of concordance was shown between professional benefits cited and professional development indicators within UK work force development frameworks. A theoretical trajectory from volunteer experience to UK service delivery outcomes was demonstrated in most areas, but not all. 32% of sources cited costs, yielding 15 initial codes which were grouped into 5 domains: financial; reputational; health & security; loss of staff; and opportunity costs.

**Conclusions:**

There is little published or unpublished literature on the impact of volunteering within health partnerships to British individuals, institutions or the UK. The existing evidence base is descriptive and focuses on the benefits of volunteering. More work is required to quantify the costs and benefits of volunteering within health partnerships for individuals and institutions, and the associated challenges and barriers. Despite these limitations our analysis suggests that there is a strong theoretical argument that the skills acquired through volunteering are transferable to service delivery within the NHS and that the benefits to individuals and institutions could be maximised when volunteering is formally embedded within continuing professional development processes.

## Background

Partnerships to share learning and resources between UK institutions and collaborators in Low and Lower- Middle Income Countries are one model to improve health care delivery [1]. It has been proposed that such links promote genuine understanding and respect for different societies and cultures [2], offer a more sustainable, locally-led model of development, build capacity and strengthen health systems in developing countries [3].

Since the publication of the Crisp report [1] in 2007, there has been increasing governmental support for health partnerships [4,5] and there are now over 100 involving UK institutions [3].

Evaluation of health partnerships has largely focused on the benefits to developing countries, which are thought to include greater access to financial and scientific resources, and capacity-building for health-care delivery and research [2]. Benefits to the UK have received less attention, and while many claims have been made, there has been no review of the literature to support these. The evaluations that have been published have focused on the benefits with less emphasis on the costs (a term we utilise to mean disbenefits: economic, personal or professional) and with little attempt at synthesis [6]. An analysis of the costs is essential as the overall impact of an intervention can be considered as the total benefits or advantages after taking into account the total costs or disadvantages. Thus the failure of existing literature to consider costs has prevented a comprehensive evaluation of the impact of partnerships.

We set out to draw together the published research and grey literature on the benefits and costs of institutional health links to UK partners. We sought to identify benefits at all levels (individual, institutional and national) and to show how these are linked. We hope that this work will help to structure future evaluations of health links.

## Methods

### Study design and sample

Our systematic review comprised both peer-reviewed and grey literature, and was conducted in November 2012. Grey literature was included because there are few peer-reviewed papers in this area, and unpublished policy documents and project reviews are likely to play a key part in this emerging and complex field [7-9].

The inclusion criteria were agreed by the project team on the basis of a preliminary review of definitions of health partnerships. This suggested that health partnerships might impact at individual, institutional and national levels and impacts at all levels were included to gain as thorough an evaluation as possible.

Our inclusion criteria were published or grey literature on health partnerships where:

1) The link is between two and only two organisations (as links between multiple institutions have been shown to differ significantly in aims and impact) [2]

2) One of which must be in the UK and one of which must be in a lower income or lower-middle income country

3) The relationship extends beyond a single event (since the concept of partnership implies a relationship that extends through time)

4) Activities have a health focus

5) UK participants are volunteers i.e. not in receipt of a full salary (with no specific criteria as to type of volunteering defined)

6) There is reference to benefits or costs in at least one of the three levels (individual, institutional or national)

For each element of the search one team member reviewed all initial results, to ensure that they met the inclusion criteria and 20% of each set of results were cross-checked by a different team member. Any disagreements about application of criteria or extraction of data were resolved through negotiated consensus.

### Data sources and study selection

#### Peer-reviewed literature

12 electronic databases were searched for published literature using a standard set of search terms about the impact of volunteering within health links to individuals, institutions and the UK, and any literature published since the earliest date indexed in each database to the current date was included. Search questions can be found in Additional file 1: Appendix 1.

The 12 databases were: PUBMED, Cochrane Economic Evaluations, Health Management Information Consortium, Health Business Elite, SCOPUS, Web of Knowledge/Social Sciences Citation Index, PsycINFO, CINAHL, AMED, International Bibliography of Social Sciences, Social Services Abstracts and Sociological Abstracts, Global Health and JSTOR.

The titles and abstracts of all the initial search results found from the electronic databases were screened and all articles unrelated to health partnerships were excluded (for detailed breakdown of findings see Additional file 1: Appendix 2). All articles retained were then screened again to determine if they met our inclusion criteria.

#### Unpublished literature

One hundred and twenty websites were identified by reviewing the first 30 hits for each of four search terms through Google. Search questions can be found in Additional file 1. A four step website search was then undertaken to find relevant unpublished literature: First, an initial decision was taken as to whether the website contained any information on international health partnerships, through an evaluation of the landing page, home page, site map and contents page (where available). Then, if the website contained relevant information, landing pages of each section of the website were screened to determine whether information relating to health partnerships was only present in one section, or in multiple sections. If only one section contained relevant information then only this section would be screened; if multiple sections were relevant then the whole website was screened. Next, all web pages and documents containing information about health partnerships were extracted, except for project databases and editions that were produced regularly (e.g. newsletters) which were excluded. Lastly literature which did not meet the inclusion criteria was excluded.

Communicating with experts in the field has been established as another means of locating grey literature [10]. Two experts, Lord Nigel Crisp (Independent Crossbencher and Author of the Crisp Report, 2007 [1]), and Graeme Chisholm (Volunteer Engagement Manager, THET: The Tropical Health and Education Trust, responsible for working with individuals participating in health partnerships) were emailed explaining the aims and purpose of the review and requesting information about any relevant past or current projects about which they might be aware. The only data collected from these conversations were lists of possible sources of information. Both responded with many useful and relevant sources of information but only one of these met all of our inclusion criteria (a set of Voluntary Service Overseas (VSO) web pages).

#### Citation-mapping

Following collation of all documents that met the inclusion criteria (both peer-reviewed and grey literature), all bibliographies were reviewed and all references that were of potential relevance were assessed against the inclusion criteria. Finally, all sources were assessed for the level of evidence they provided, according to the scoring system recommended by Benzies et al., 2006 [9] which can be found in Additional file 1. This system describes five levels of evidence, based on the rigor of the research methodology, ranging from level I (randomized control trials) to level III (non-randomized, controlled or cohort studies, case series, case controlled studies, or cross-sectional studies) to level V (the opinion of informed individuals).

### Data extraction and analysis

All documents that met the inclusion criteria were scanned in their entirety, and all text that referenced benefits or costs at any of our three levels (individual, institutional and national) was extracted for analysis.

Since the majority of studies found during the search were qualitative in nature, thematic analysis was chosen to synthesise the findings. Thematic synthesis is a method frequently used to analyse data in primary qualitative research, which has three stages which may overlap: the coding of text ‘line-by-line’; the creation of ‘descriptive themes’; and the generation of ‘analytical themes’ [11].

All textual data from websites and papers was therefore extracted into an excel spreadsheet and separated into ‘line-by-line’ content. Through a process of reading and re-reading we generated a comprehensive list of codes to map all recorded costs and benefits to fully describe the data in these terms. The individual costs and benefits were then grouped into descriptive themes that grouped similar outcomes. To understand the implications of this description of costs and benefits we interrogated our data further to draw out their implications for professional development and potential impact on clinical practice and service development. In this process we attempted to interpret and ‘go beyond’ initial data to form new constructs, explanations or hypothesis [12]. The decisions in all, but particularly the third stage, are dependent on the judgement and views of the reviewer, so decisions made at all three stages were discussed and agreed by all team members.

Since we considered benefits at three levels (individual, institutional and national) we sought frameworks to show how these were linked. To link individual benefits to institutional benefits we mapped those identified from the literature review against five frameworks for skill acquisition in the NHS. These frameworks were chosen to cover professional development of doctors of all specialties (the Academy of Royal Colleges’ [13] and NHS Leadership and Development frameworks [14]) and all other NHS workers (the Knowledge and Skills framework [15]), and to cover all stages of careers, from minimum standards at qualification (the General Medical Council’s Good Medical Practice [16]), to skills expected at consultant level and beyond (The Continuing Professional Development curricula [17]). The focus upon frameworks relating to medical careers was chosen as all partnerships studied had included medical professionals, whereas no other health professional was universally represented.

To link individual benefits for health professionals to outcomes for those using UK health services we used a modified version of a framework developed by Wales for Africa [18].

## Results

The electronic databases of published literature returned 43 hits, including duplicates (Additional file 1). Eight articles were retained after comparison to inclusion criteria, only five of which contained data relevant to the evaluation of the UK partner. An additional four published articles were found through the citation mapping of both grey and peer-reviewed literature, bringing the total number of published articles for data extraction to 9.

The grey literature search (through websites and experts in the field) returned forty-five unique sources, of which twenty-seven met all inclusion criteria. An additional five pieces of literature were found through the citation mapping of both grey and peer-reviewed literature, bringing the total number of pieces of grey literature for data extraction to thirty-two.

A total of forty-one sources (nine peer-reviewed and thirty-two grey literatures) were retained for data extraction. Of these, most were of a poor quality; only 17% contained any attempts at qualitative or quantitative data analysis. No sources reached the top two levels of evidence (randomized control trials) and only 12% of the 41 sources fell into level 3 (“based on non-randomized, controlled or cohort studies, case series, case controlled studies, or cross-sectional studies” [9]). Furthermore 49% of the literature failed to reach even the lowest level of evidence and 44% of the grey literature failed to provide any data to back up assertions made. For more details see Tables 1 and 2.

**Table 1:** Summary of published literature

**Study**	**Date**	**Journal**	**Author**	**Level of evidence**
Hands Across the Equator	1988	BMJ	Wood JB, Hills EA	VI
Hands Across the Equator: 8 years on	1994	BMJ	Wood JB, Hills EA, and Keto FJ	VI
Research into practice: 10 years of international public health partnership between the UK and Swaziland	2010	Journal of Public Health	Wright J, Walley J, Philip A, Petros P, Ford H	Vb
International health links: an evaluation of partnerships between health-care organizations in the UK and developing countries	2006	Tropical Doctor	Baguley D, Killeen T, Wright J	III
International Health Links movement expands in the United Kingdom	2010	International Health	Leather A, Butterfield C, Peachey K, Silverman M, Sheriff R	Vb
Global health partnerships: leadership development for a purpose	2009	Leadership in Health Services	Hockey P, Tobin A, Kemp J, Kerrigan J, Kitsell F, Green P, Sewell A, Smith C, Stanwick S, Lees P	Vb
NHS Links: a new approach to international health links	2005	BMJ Careers	Sloan J, Wright J, Silverman M	Vb
UROLINK – benefits for trainees from both sides	2002	British Journal of Urology International	Gujral S, Nassanga R	Vb
Twinning: the future for sustainable collaboration	2002	British Journal of Urology International	MacDonagh R, Jiddawi M, V. Parry V	Vb

**Table 2:** **Summary of the Grey Literature (see Additional file ****1****: Appendix 3 for definitions of each category and Additional file ****1****: Appendix 4 for a detailed breakdown of grey literature)**

**Organisational affiliation of author**	**Number of documents**	**Category of literature**	**Number of documents**	**Level of evidence**	**Number of documents**
National Governmental Organisation	4	Policy Document	4	Level I	0
National Non-Governmental	14	Guidance Document	3	Level II	0
Sub National Governmental Organisation	4	Evaluation Document	6	Level III	4
Sub National Non-Governmental Organisation	3	Project Announcement or Project Report	5	Level IV	2
Academic Institution	5	Conference Report	2	Level V (a)	2
Individual Health Link	2	Presentation	3	Level V (b)	6
		Webpages	5	Ungraded (Level VI)	18
		Press Release	4		
**Total Number of Studies:**	**32**	**Total Number of Studies:**	**32**	**Total Number of Studies:**	**32**

### Synthesis of published and grey literature

We completed a thematic analysis of the qualitative data from our review, the results of which can be seen in Additional file 1: Appendix 5. The majority of our sources (95%) cited benefits and we developed 40 independent initial codes of benefits to individuals.

These benefits to individuals were assigned to seven domains, six of which were skills-based: clinical skills, management skills, communication and teamwork, patient experience and dignity, policy, academic skills, and the last of which related to personal satisfaction and interest, as shown below in Table 3. Additional file 1: Appendix 6 categorises these individual benefits as knowledge, attitudes and skills.

**Table 3:** Grouping codes into domains for individual benefits

**Domain**	**Benefit to individual**	**Initial codes**
**Clinical skills**	Tropical Diseases	Learning about tropical conditions
	Clinical Skills	Able to manage without technology
**Management skills**	Innovation in healthcare delivery and	Creative thinking, resourcefulness, innovation, problem-solving
	use of resources	
	Ability to Cope in Different Environments	Adaptability, flexibility, ability to cope in pressurised environment, ability to cope with complexity
	Prioritisation of Limited Resources	Resource management
	Self–Understanding	Self-awareness, self-reliance, humility, understanding of own limits
	Leadership and Management	Leadership and management
**Communication and teamwork**	Improved skills of negotiation with multiple stakeholders	Diplomacy
	Team-working	Cross-sectoral teams, multi-disciplinary working
	Increased appreciation of and skills in maintaining of relationships	Build productive relationships, new friends, value of relationships
	Languages	Opportunity to learn and use languages
**Patient experience and dignity**	Greater appreciation of factors influencing health in other countries	Understanding of the global context, understanding of needs of developing countries
	Increased knowledge and appreciation of other cultures	Knowledge of other cultures, understanding of people from other countries
**Policy**	Understanding of other health systems	Ability to work in other health systems
	Perspective on UK problems	Appreciation of NHS, perspective on UK problems
	New Ideas	Appreciation of value of new ideas, openness to new ideas
**Academic skills**	Education, Training and Research	Training delivery & research skills, understanding of how to target training, learning to apply for grants, utilising policy skills, research ideas, opportunities & interest
**Personal satisfaction and interest**	Lifelong Interest in Global Health & Development	Lifelong interest in global health and development
	Personal Satisfaction	New relationships and friends, learning languages, delivering training

The evidence for benefit on institutional level, and national level in particular was weak. However, we extracted 10 benefits to institutions, and 10 national benefits. Some of these (such as reputational development and demonstrating Corporate Social Responsibility) seemed to arise directly from the existence of a link, independently of the benefits derived by individuals participating in the link, whilst others (such as staff with an understanding of the global context) seemed to arise as a result of individuals who were part of the institution or national workforce participating in links. The number of individuals participating in a link is very small percentage of the workforce. Therefore the perception that benefits such as “reduction of waste within the NHS” arise is based on the (untested) hypothesis that link participants are able to significantly change institutional and national systems on their return, in order to impact upon service outcomes. 13 sources (32%) cited costs and we developed independent initial codes for costs which were grouped into 5 domains; financial, loss of staff, reputational, health and security and opportunity costs, as demonstrated in Table 4. Many of these costs, particularly with regards to loss of staff and the opportunity costs, arose from the adhoc relationship of partnerships work to other professional activities; for example the difficulties of organising cover for those participating in links was prominently cited as a cost of volunteering.

**Table 4:** Overview of costs

**Domain**	**Initial codes**
Financial	Financial cost
Loss of Staff	Loss of staff from other areas of work, Imposing upon others when finding cover, Challenges of organising cover, Trained staff leaving their post following links
Reputational	Negative perception of the UK Institution where links are run badly, Negative perception of the UK where links are run badly
Health and Security	Accidents/Injury, Management of security risks, Exhaustion/Burnout/Stress, Culture shock
Opportunity	Staff distracted from areas of UK work, Neglect of relationships/Burden of Family or friends, Loss of annual leave, Negative effects on career, Opportunity costs

### Analysis of results

Some individual benefits such as improved motivation, team-working, and an understanding of patients from different cultures and backgrounds were considered to contribute to UK health workforce development. To investigate this link we mapped the benefits found in the six skills-based domains against five frameworks used by institutions to structure workforce skills and development (building on previous work in this field [19]). The results of this process are shown in Table 5 which documents the close relationships between the benefits which our review suggests arise from participating in health links and the attributes that UK health institutions are seeking to develop within the health care workforce.

**Table 5:** Mapping individual benefit domains onto existing professional development frameworks

**Domain**	**Benefits identified by this literature review**	**Knowledge & skills framework (KSF)**	**Common competencies for doctors (Academy of royal medical colleges)**	**Continuing professional development**	**NHS leadership framework**	**GMC good medical practice**
**Clinical skills**	Clinical skills, managing tropical diseases, ability to cope in different environments	Quality, personal development	Basic clinical competencies	Knowledge, Skills and Performance	Demonstrating personal qualities	Good clinical care
**Management skills**	Innovation, prioritisation of limited resources	Health, safety, security	Patient safety, Legal & ethical aspects of care, Management and leadership	Safety and Quality	Managing services	Maintaining good medical practice
**Communication and teamwork**	Teamwork, relationships, diplomacy, languages	Communication, people development	Communication	Communication, Partnership and Teamwork	Working with others	Working with colleagues
**Patient experience and dignity**	Understanding of different cultures, understanding of global health	Equality and diversity	Personal attitudes & behaviour, patient safety	Maintaining Trust	Demonstrating personal qualities	Relationship with patients
**Service/policy development & implementation**	Understanding of other health systems, diplomacy	Service improvement			Improving services, setting direction, creating vision, delivering strategy	
**Academic skills**	Education, research		Standards of care and education			Teaching, training, appraising and assessing

The Knowledge and Skills framework, of which core dimensions are considered in Table 5 is further expanded in table Additional file 1: Appendix 7 to detail 29 skill dimensions each with four levels of descriptor. Benefits from health partnerships engagement were shown to map directly onto the most advanced descriptor of each dimension. This suggests any member of the NHS workforce could gain or improve skills from volunteering through a health link in a way which is recognised to be of benefit to individuals in their NHS jobs, institutions and ultimately patient care.

**Figure 1:**
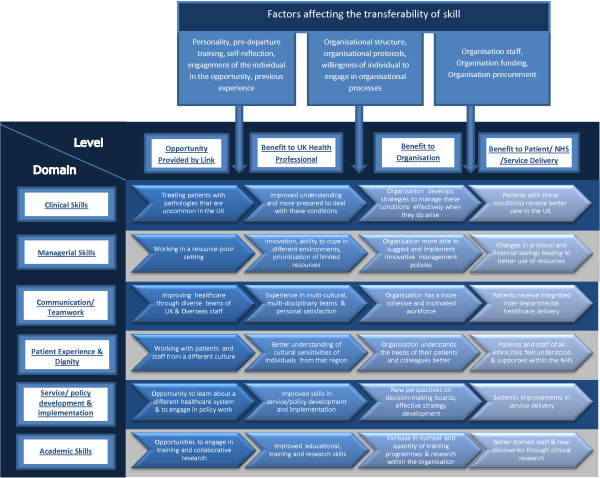
Transmission of health partnership opportunities into improvements in service delivery and patient experience.

To illustrate this relationship further and to extrapolate it to health care delivery we further developed a framework used by Wales for Africa and published on their website [18]. This shows how the experience of volunteering could impact on individuals, institutions and health care service users, in all six skills-based domains as shown in Figure 1. However, not all new skills were relevant to UK healthcare delivery; such as learning to cope without the imaging equipment available in the UK.

### Key findings and their significance

Key findings included:

● There is little peer-reviewed and grey literature published on this topic, and that which has been published largely fails to meet high standards of formal academic rigor.

● Much of the literature is written by those involved in facilitating or advocating for links, with few external evaluations.

● With the limitations described above, the existing literature suggests that the benefits of health partnerships outweigh costs.

● The individual benefits fell into 7 domains, 6 of which were skills-based, and are closely linked to existing UK frameworks to structure health workforce development.

● We extracted 10 benefits to institutions and 10 national benefits.

● Institutional and national benefits could be seen to arise both from the existence of links, and as a result of individuals from the UK workforce benefitting through link experiences.

● There is a theoretically clear trajectory that links volunteer experience to improved health service delivery for some categories of benefits (e.g. leadership) but not others (e.g. working in situations without access to clinical investigations).

● Five cost domains were identified: financial, loss of staff, reputational, health and security and opportunity costs.

● More data is needed on the relative impact of costs and benefits for individual volunteers or within an individual link, and the factors influencing these.

## Discussion

### Limitations of our study

We reviewed the limited literature available on the UK benefits of health partnerships and identified a range of costs and benefits. There was wide variation between the links reviewed, making it difficult to draw universal comparable conclusions. Standardised, valid and reliable evaluation tools should be used in the future to enable comparison between links, and to facilitate aggregation of data between different trips within a partnership, and different partnerships.

Our analysis showed that the impact of a link is closely related to how effectively it is run, and this was a key confounding factor. For example, the cost of a negative perception of the UK institutions or UK was raised by southern partners [20,21] who commented that where links are poorly run or have prominent power imbalances they have in some instances harmed the UK reputation. However, where run appropriately links have been seen to enhance the reputation of the UK; this variety within links and their impacts was noted in the Department of Health’s evaluation of links (2008) [5].

We have shown that the skills developed through volunteering in a link are considered valuable skills for NHS employees according to numerous professional development frameworks. However, it is impossible to map the level of skills attained against the level required in these frameworks as existing studies rely heavily on volunteers reports of subjective improvement, untested against such frameworks. Thus, further work is required to develop a structure for optimising goal setting and measuring skill development during health partnership placements.

The literature suggests that health partnerships could have an important positive impact for UK health professionals and institutions. However some skills learnt during volunteering are more transferable than others, or transferable in some contexts but not others. For example, experience of tropical diseases might be only relevant to those who work in the UK with populations where such diseases are common. More careful analysis of the factors affecting translation of experience to impact on clinical practices and services at each stage is required in order to modify them so as to maximise the positive impacts of health partnerships to the UK. Some possibilities have been shown in Figure 1.

#### Limitations of the literature reviewed

Limitations of studies reviewed included small sample sizes, evaluation by those involved in the partnership, retrospective reporting, and measurement of outcomes rather than impact. Additionally, all studies failed to utilize control groups or comparison with other activities that may offer also individual, institutional or national benefits e.g. UK based medical leadership programmes.

This review necessarily focused solely on one type of volunteering in Low or Lower- middle income countries, limiting the scope of its impact. Health partnerships represent only a fraction of total volunteering for global health development, which may take place through numerous other mechanisms such as non-governmental organisations (NGOs), gap year and student volunteering programmes and individual activity.

There is more work to be done to demonstrate to what extent our findings are generalizable to other volunteering programmes, and whether there are unique benefits derived from health partnerships in comparison to other models of volunteering. The literature we reviewed encompassed links providing of clinical services, training, and other support services, professionals ranging from healthcare to managerial departments and beyond, and both direct volunteering in the UK and volunteering through on-line mentorship and online teaching. Thus the heterogenous nature of health partnerships implies both that there is more work to be done regarding stratification of different forms of partnership, and that there may be many areas of relevance and interest for other volunteering programmes.

### Recommendations for Healthcare policy

The findings of this review and their application to key frameworks suggested strategies with potential to maximise the benefits of health links. Since many costs appear to result from the ad hoc relationship between partnership work and other professional activities one such strategy would be the formal integration of volunteering within partnerships into NHS Continuing Professional Development (CPD) frameworks (as previously explored by Longstaff B). This would potentially release study leave funding to support volunteering within links, minimising additional costs to individuals and the NHS, making it easier to organise cover, and therefore reducing many of the ‘loss of staff’ and ‘financial’ costs raised. Volunteering at least partially within study leave would protect volunteers’ annual leave, and thus help prevent burnout. Benefits of the strategy would include transferring some financial burden from the individual to the institution, which might in turn increase engagement, formal recognition of the skills gained by individuals through link participation, and justification of investment of NHS resources in partnerships. The Welsh NHS has already taken this step [22] and the impact of this scheme needs thorough evaluation including cost effectiveness analysis.

### Recommendations for future data collection and evaluation

More data is needed to quantify the benefits identified and to balance these against the costs. Quantification of benefits to date [5] is entirely based on self-reporting, and on ordinal scales, providing weak grounds for comparison. Costs have only been evaluated in the financial domain and estimates of the cost of the trips overseas for an average link, vary from £10,000 - £20,000 per annum [1] to significantly higher figures [5]. Even where objective measures of costs (or disadvantages) do exist, it is challenging to compare these between the different domains identified in our analysis.

Our research clearly demonstrated the heterogeneous nature of health partnerships. New standardised evaluation tools should distinguish between the different benefits and costs experienced by different health professionals with different levels of experience in different links, thus allowing appropriately targeted future funding and personal support.

Comparison of engagement in links to alternative methods of health workforce development would enable effective comparison of learning outcomes and costs to the institution. Thus future attempts at evaluation should aim to gather baseline measures prior to engagement in a link, and to incorporate more objective, independently-assessed measures of improvement of practice in the UK. These could be based around existing competency frameworks, and existing initiatives [23,24] should be encouraged and shared. Such attempts should ideally be conducted alongside measuring benefits to the partner country, to reduce the risk of participants viewing volunteering as principally an activity for self-development, potentially at the cost of the ‘recipient’ partner.

There is a real need to develop standardised methods of data collection and reporting in this area (as previously explored by Syed et al.) to enable greater understanding of health innovation diffusion and how developed countries can benefit from the work of developed countries, both in the field of health links and beyond [25].

## Conclusions

Our review suggests that health partnerships benefit individuals, institutions, and the UK. Benefits can be mapped to improved professional skills, and by inference improved patient care, and to measurable professional development outcomes.

However the evidence to support these claims is still limited. In 2008 the Department of Health stated “there is still insufficient understanding on the impact and benefit of these links on the UK and developing partners” [4]. Unfortunately, five years on, this is still the case. Valid and reliable evaluation of the benefits and costs to the UK partner is an essential and as yet largely unrealised component of running a link. THET and the Health Partnerships Scheme have a critical role to play in supporting the development and application of standardised evaluation practices. Lack of both financial and human capacity is often cited as a reason not to evaluate, and an emphasis is often placed on evaluation of overseas impact.

However, evaluation of UK impact is equally important if health links wish to continue to gain support. To continue to expand, and to receive funds from publicly-funded institutions, links must be accountable to stakeholders and tax-payers. To be seen not just as part of a corporate social responsibility programme, but firmly embedded in the process of delivering high-quality health education and services, partnerships must demonstrate that they are improving practice in the UK as well as overseas. Furthermore, development of an evidence base to demonstrate that participation in health links is as effective or more effective than other UK-based methods of achieving CPD goals is essential if the case is to be made for the much-needed greater financial investment in health partnerships.

Finally, effective evaluation would allow individual links to monitor progress, and enable comparison between links, allowing examples of best practice to be gathered and shared. Valid data in this area would allow enhancement of benefits and minimisation of costs, and thus has the potential to hugely improve health partnerships and to upscale involvement across the UK, and indeed internationally.

## Competing interests

The authors declare that they have no competing interests to declare.

## Authors’ contributions

FAEJ participated in the design of the study, searched for unpublished literature, extracted and analysed data and drafted the majority of the manuscript. DPHK participated in the design of the study, searched for published literature and contributed to drafting the manuscript. VFES mapped benefits onto professional development frameworks and contributed to drafting the manuscript. PB conceived of the study, and provided detailed oversight of study design and data analysis. All authors participated in the coordination of the review and read and approved the final manuscript.

## Supplementary Material

Additional file 1: Appendix 1 Screening Questions and Terms. **Appendix 2.** Overview of Results from Search Engines. **Appendix 3.** Details of Categorisation of Grey Literature. **Appendix 4.** Detailed Summary of Grey Literature. **Appendix 5.** Descriptive Coding Layers. **Appendix 6.** Descriptive Coding of Individual Benefits. **Appendix 7.** Detailed Mapping of Individual Benefit Domains onto the Knowledge & Skills Framework’s most advanced descriptor (Level 4). Click here for file

## References

[B1] Crisp N Global health partnerships: the UK contribution to health in developing countries http://www.idcsig.org/Crisp%20Report.pdf

[B2] The Royal College of Physicians and the Academy of Medical Sciences Building institutions through equitable partnerships in global health http://www.universitiesuk.ac.uk/Publications/Documents/BuildingInstitutionsGlobalHealth.pdf

[B3] NHS and Department of Health The Framework for NHS Involvement in International Development http://www.thet.org/hps/resources/publications/the-framework-for-nhs-involvement-in-international-development/at_download/publicationFile

[B4] Department of Health Global health partnerships: the UK contribution to health in developing countries - the Government response http://webarchive.nationalarchives.gov.uk/20080814090248/dh.gov.uk/en/Publicationsandstatistics/Publications/PublicationsPolicyAndGuidance/DH_065374. Accessed 04/09/13

[B5] Department of Health Evaluation of links between North and South healthcare organisations http://www.build-online.org.uk/documents/Evaluation%20of%20links%20between%20N%20and%20S%20healthcare%20orgs%20-%20DFID%202008-1.pdf. Accessed 04/09/13

[B6] Smith C The role of health links in international development: the need for greater evidence? Trop Doct 2012 42 2 65 66 10.1258/td.2011.110381 22431819

[B7] Berman Y INFUSE [Information Uses in Social Welfare]—Delineation of a grey document Eurosocial Newsletter 1992 59 39 43

[B8] Mays N Pope C Popay J Systematically reviewing qualitative and quantitative evidence to inform management and policy-making in the health field J Health Serv Res Policy 2005 10 1 6 20 16053580 10.1258/1355819054308576

[B9] Benzies KM Premji S Hayden KA Serrett K State-of-the-evidence reviews: advantages and challenges of including grey literature Worldviews Evid Based Nurs 2006 3 2 55 61 10.1111/j.1741-6787.2006.00051.x 17040510

[B10] Shekelle PG Morton SC Suttorp MJ Buscemi N Friesen C Challenges in systematic reviews of complementary and alternative medicine topics Ann Intern Med 2005 142 12 1042 1047 15968028 10.7326/0003-4819-142-12_part_2-200506211-00003

[B11] Thomas J Harden A Methods for the thematic synthesis of qualitative research in systematic reviews BMC Med Res Methodol 2008 8 12 10.1186/1471-2288-8-12 18616818 PMC2478656

[B12] Britten N Campbell R Pope C Donovan J Morgan M Pill R Using meta ethnography to synthesise qualitative research: a worked example J Health Serv Res Policy 2002 7 4 209 215 10.1258/135581902320432732 12425780

[B13] Academy of Royal Colleges Common Competencies Framework for Doctorshttp://www.aomrc.org.uk/education-a-training/curriculum-and-framework/framework.html. Accessed 04/09/13

[B14] NHS Leadership Academy Clinical Leadership Competency Framework http://www.leadershipacademy.nhs.uk/wp-content/uploads/2012/11/NHSLeadership-Leadership-Framework-Clinical-Leadership-Competency-Framework-CLCF.pdf. Accessed 04/09/13

[B15] Department of Health The NHS Knowledge and Skills Framework and the Development Review Process http://www.msg.scot.nhs.uk/wp-content/uploads/KSF-Handbook.pdf

[B16] General Medical Council: Good Medical Practicehttp://www.gmc-uk.org/guidance/good_medical_practice.asp

[B17] General Medical Council Good Medical Practice Framework for appraisal and revalidation London, General Medical Councilhttp://www.gmc-uk.org/doctors/revalidation/revalidation_gmp_framework.asp

[B18] The Benefits of Health Linkinghttp://www.wales.nhs.uk/sites3/page.cfm?orgid=834&pid=63509

[B19] Longstaff B Can partnerships be truly mutually beneficial? What are the benefits to the NHS? http://www.wales.nhs.uk/sites3/Documents/834/Brenda%20Longstaff.pdf

[B20] Sewankambo KN The Value and Challenges of Institutional Partnerships in Global Health: A view from the Southhttp://www.rcplondon.ac.uk/sites/default/files/nelson_sewankambo_-_the_value_and_challenges_of_institutional_partnerships_2.pdf

[B21] Holm J Malete L The Asymmetries of University Partnerships between Africa and the Developed World http://www.thet.org/wp-content/uploads/2010/04/Asymmetries-of-University-Links.pdf

[B22] Leather A Butterfield C Peachey K Silverman M Sheriff R International Health Links movement expands in the United Kingdom International Health 2010 2 3 165 171 10.1016/j.inhe.2010.04.004 24037696

[B23] The International Health Links Centre (IHLC) and The London Deanery GP Trainee Time Out of Programme (OOP): Exploration of Impact on Skillshttp://www.londondeanery.ac.uk/general-practice/files/gp-trainee-oop-lstm-evaluation0311.pdf

[B24] Hockey P Tobin A Kemp J Kerrigan J Kitsell F Green P Sewell A Smith C Stanwick S Lees P Global health partnerships: leadership development for a purpose Leadersh Health Serv 2009 22 4 306 316 10.1108/17511870910996105

[B25] Syed S Dadwal V Rutter P Storr J Hightower J Gooden R Carlet J Nejad S Kelley E Donaldson L Pittet D Developed-developing country partnerships: benefits to developed countries? Glob Heal 2012 8 17 10.1186/1744-8603-8-17 PMC3459713 22709651

