# Supplementary material for: Do health partnerships with organisations in lower income countries benefit the UK partner? A review of the literature
Source: Global Health. 2013 Aug 30;9:38. doi: 10.1186/1744-8603-9-38 (PMC3766651; doi:10.1186/1744-8603-9-38)
Supplement: Additional file 1: Appendix 1 — Screening Questions and Terms. Appendix 2. Overview of Results from Search Engines. Appendix 3. Details of Categorisation of Grey Literature. Appendix 4. Detailed Summary of Grey Literature. Appendix 5. Descriptive Coding Layers. Appendix 6. Descriptive Coding of Individual Benefits. Appendix 7. Detailed Mapping of Individual Benefit Domains onto the Knowledge & Skills Framework’s most advanced descriptor (Level 4). [file 1744-8603-9-38-S1.docx]

**Additional file 1: Appendix 1: Screening Questions and Terms**

**Database Search Terms & Search Question***We searched for peer-reviewed literature in 12 electronic databases (PUBMED, Cochrane Economic Evaluations, Health Management Information Consortium, Health Business Elite, SCOPUS, Web of Knowledge/Social Sciences Citation Index, PsycINFO, CINAHL, AMED, International Bibliography of Social Sciences, Social Services Abstracts and Sociological Abstracts, Global Health and JSTOR), including any literature published since the earliest date indexed in each database to the current date.*

**Search Terms Table**

| **Cost/Benefit** | **What** | **Who** | **Where (Home)** | **Where (Away)** |
| --- | --- | --- | --- | --- |
| Impact | “Health Link” | Doctor | UK | Overseas |
| Impacts | “Health Links” | Doctors | “United Kingdom” | Foreign |
| Benefit | “Health Partnership” | Nurse | Britain | International |
| Benefits | “Health Partnerships” | Nurses | England | “Low Income Countries” |
| Cost |  | “Health Professional” | Scotland | “Low Income Country” |
| Costs |  | “Health Professionals” | Wales | “Lower Middle Income Countries” |
| Outcome |  | University | “Northern Ireland” | “Lower Middle Income Country” |
| Outcomes |  | Universities | British | “Developing Countries” |
| Evaluate |  | Hospital | English | “Developing Country” |
| Evaluation |  | Hospitals | Scottish | “Global South” |
| Evaluations |  | “Health Institution” | Welsh |  |
|  |  | “Health Institutions” | “Northern Irish” |  |
|  |  | NHS |  |  |

**Search Question**

*(Impact OR Impacts OR Benefit OR Benefits OR Cost OR Costs OR Outcome OR Outcomes OR Evaluate OR Evaluation OR Evaluations)* ***AND*** *("Health Link" OR "Health Links" OR "Health Partnership" OR "Health Partnerships")* ***AND*** *(Doctor OR Doctors OR Nurse OR Nurses OR "Health Professional" OR "Health Professionals OR University OR Universities OR Hospital OR Hospitals " OR "Health Institution" OR "Health Institutions" OR NHS)* ***AND*** *(UK OR "United Kingdom" OR Britain OR England OR Scotland OR Wales OR "Northern Ireland" OR British OR English OR Scottish OR Welsh OR "Northern Irish")* ***AND*** *(Overseas OR Foreign OR International OR "Low Income Countries" OR "Low Income Country" OR "Lower Middle Income Countries" OR "Lower Middle Income Country" OR "Developing Countries" OR "Developing Country" OR "Global South")*

**Website Search Terms***We searched 120 websites, searching the first 30 for each of four search terms found below through google.*

| **Search Number** | **Search Term** |
| --- | --- |
| 1 | UK Health Partnerships |
| 2 | UK Health Links |
| 3 | UK Institutional Health Partnerships |
| 4 | UK Institutional Health Links |

**Additional file 1: Appendix 2: Overview of Results from Search Engines**

| **Database** | **Number of hits** | **Number relevant** | **Relevant Paper 1** | **Relevant Paper 2** | **Relevant Paper 3** | **Relevant Paper 4** |
| --- | --- | --- | --- | --- | --- | --- |
| Medline (Pubmed) | 14 | 2 | Wright et al | Baguley et al |  |  |
| Cochrane Economic Evaluations | 0 | - |  |  |  |  |
| Health Management Information Consortium | 2 | 2 | Wright et al | Sloan et al |  |  |
| Health Business Elite | 0 | - |  |  |  |  |
| Scopus | 4 | 4 | Leather et al | Wright et al | Hockey et al | Baguley et al |
| Web of Knowledge | 3 | 2 | Wright et al | Baguley et al |  |  |
| PsychINFO | 0 | - |  |  |  |  |
| CINAHL | 1 | 1 | Wright et al |  |  |  |
| AMED | 0 | - |  |  |  |  |
| International Bibliography of Social Sciences, Social Services Abstracts and Sociological Abstracts | 15 | 0 |  |  |  |  |
| Global Health | 4 | 3 | Leather et al | Wright et al | Baguley et al |  |
| JSTOR | 0 | - |  |  |  |  |

**Additional file 1: Appendix 3: Details of Categorisation of Grey Literature

Category of Author***A note: Sub-national government refers to the Welsh assembly. Sub-national NGO refers to Wales to Africa.* **Category of Literature**

| **Category** | **Description** |
| --- | --- |
| *Policy   Document* | May overtly declare itself to be policy. Authored by respected authorities or that of an expert   committee. Produced as a considered consensus of such a body. Guides strategic direction. |
| *Guidance   Document* | May overtly declare itself to be guidelines, or to aim to support or facilitate actions. Produced as   a considered consensus. Focuses on practical implications. |
| *Evaluation   Document* | May overtly declare an aim to evaluate. Always includes data, and should focus around and  make significant attempts at a reasonable level of analysis. |
| *Project Announcement   or Report* | May overtly declare an aim to inform. Sets out what is happening, primarily for funder or to   attract possible stakeholders. Minimal data and low levels of analysis. |
| *Conference   Report* | Must overtly declares itself to be a conference report. Only included if it includes data   which cannot otherwise be found. |
| *Presentation* | A powerpoint or video. Only included if it includes data which cannot otherwise be found. |
| *Webpages* | Found on a website, independent of other documentation. Only included if it includes data which   cannot otherwise be found. |

**Level of Evidence**

| **Level of Evidence** | **Description** |
| --- | --- |
| Level I | Based on randomized, controlled trials (or meta-analysis of such trials) of adequate size to ensure a  low risk of incorporating false-positive or false-negative results |
| Level II | Based on randomized, controlled trials that are too small to provide Level I evidence. These may  show either positive trends that are not statistically significant or no trends and are associated with  a high risk of false-negative results |
| Level III | Based on non-randomized, controlled or cohort studies, case series, case controlled studies, or  cross-sectional studies |
| Level IV | Based on the opinion of respected authorities or that of an expert committee as indicated in  published consensus conferences or guidelines |
| Level V (a) | Based on the opinion of those individuals who have knowledge in one particular field and are  applying that knowledge to another field; or summarizes the collective wisdom or experiences of  others in the field |
| Level V (b) | Based on the opinion of those individuals who have written and reviewed the guidelines, based on  their experience, knowledge of the relevant literature, and discussion with their peers |
| *Ungraded*  *(VI)* | *Does not meet the above Criteria* |

**Additional file 1: Appendix 4: Detailed Summary of Grey Literature**

| **Title/Description of Grey Literature** | **Source** | **Category of Author** | **Category of Literature** | **Level of Evidence** |
| --- | --- | --- | --- | --- |
| THET Website | Website Search 1 | National NGO | Webpages | VI |
| Global Health Partnerships: The UK contribution to health in developing countries | Website Search 1 | National Government | Policy | IV |
| Global Health Partnerships: The UK Contribution to Health In Developing Countries - the UK response | Website Search 1 | National Government | Policy | IV |
| Monitoring and evaluation for Health Links: Evaluating the impact of the Health Link on the UK Partner | Website Search 1 | National NGO | Guidance | Vb |
| The Framework for International Development | Website Search 1 | National Government | Policy | Vb |
| Developing Global Health Link Partnerships to improve Health Capacity in Developing Countries | Website Search 1 | National NGO | Evaluation | Va |
| Wales to Africa Executive Report | Website Search 1 | Sub- National Government | Evaluation | III |
| The Asymmetries of University Partnerships between Africa and the Developed World | Website Search 1 | Academic Institution | Project Report | VI |
| THET Links Manual | Website Search 1 | National NGO | Guidance | Va |
| DFID Press Release Regarding Health Partnerships | Website Search 1 | National Government | Press Release | VI |
| RCPCH Links Scheme | Website Search 1 | National NGO | Webpage | VI |
| Evaluation of links between North and South healthcare organisations (2008) | Website Search 1 | National NGO | Evaluation | III |
| Opportunities offered by participation in a Health Link | Website Search 2 | Sub- National NGO | Evaluation | VI |
| Brenda Longstaff's Benefits Presentation | Website Search 2 | Academic Institution | Evaluation | III |
| Anaesthetists selected for volunteering scheme to save lives in the world’s poorest countries (2012) | Website Search 2 | National NGO | Press Release | VI |
| The Impact of Health Links between UK Health Institutions and their Counterparts in Developing Countries | Website Search 2 | Academic Institution | Evaluation | VI |
| Links with Africa provide excellent opportunities for all who take part – Press Release | Website Search 2 | Sub- National Government | News Paper Article | VI |
| Health within and beyond Welsh borders: An enabling framework for international health engagement | Website Search 2 | Sub- National Government | Policy | Vb |
| THET March Meeting Report - Motec at Ghana-UK/THET Health Links Workshop 21st March 2007 | Website Search 2 | National NGO | Conference Report | VI |
| THET Powerpoint: Education -Health links | Website Search 3 | National NGO | Presentation | VI |
| UK benefit of the King’s –THET- Somaliland Partnership: Presented by Oliver Johnson | Website Search 3 | Academic Institution | Presentation | III |
| UK International Health Links Funding Scheme Announcement | Website Search 3 | National NGO | Programme Announcement | VI |
| An Introduction to the Vision 2020 Links Programme | Website Search 3 | National NGO | Programme Announcement | VI |
| The Value and Challenges of Institutional Partnerships in Global Health - A View from the South | Website Search 4 | Academic Institution | Presentation | VI |
| Building institutions through equitable partnerships | Website Search 4 | National NGO | Conference Report | VI |
| New UK Government Scheme to Save Lives in Malawi | Website Search 4 | National NGO | Press Release | VI |
| Voluntary Service Overseas (VSO) Website Pages | Graeme Chisholm | National NGO | Webpages | VI |
| Wales to Africa Annual Report 2011 | Citation Mapping | Sub- National NGO | Project Report | Vb |
| NHS Wales Health Links with Sub-Saharan Africa and other Developing Health Systems | Citation Mapping | Sub- National Government | Policy | Vb |
| Wales-Africa 2008 Report | Citation Mapping | Sub- National NGO | Project Report | Vb |
| Africa-Leicester Link Website | Citation Mapping | Individual Health Link | Webpages | VI |
| Ethiopia-Gwent Link Website | Citation Mapping | Individual Health Link | Webpages | VI |

| **Benefits for the Individual** | **Benefits for the Institution** | **Benefits for the UK** |
| --- | --- | --- |
| Self – Understanding | Reputational Development ’Corporate Social Responsibility' | Reputational Development |
| Innovation in healthcare delivery and use of resources | Professional development of workforce | Staff with understanding of global context |
| Ability to Cope in Different Environments | Improved motivation & cohesion of the workforce | Staff who understand patients from many backgrounds |
| Education, Research and Policy | Attraction & retention of (more/better quality) workforce | Reduction of waste within NHS |
| Languages | Staff who understand patients from many backgrounds | Experience of Tropical Diseases |
| Increased knowledge and appreciation of other cultures | New perspectives, policy & practice | Improved motivation & cohesion of the workforce |
| Greater appreciation of factors influencing health in other countries | Implementation of systemic resource-saving ideas | Attraction & retention of (more/better quality) workforce |
| Tropical Diseases | Collaborative research opportunities | Workforce appreciate NHS better |
| Clinical Skills | Increased workforce productivity | Improved patient experience |
| Team-working |  |  |
| Leadership and Management |  |  |
| Increased appreciation of and skills in maintaining of relationships |  |  |
| Prioritisation of Limited Resources |  |  |
| Improved skills of negotiation with multiple stakeholders |  |  |
| Perspective on UK problems |  |  |
| Ability to work in other health systems / understanding of other health systems |  |  |
| New Ideas |  |  |
| Personal Satisfaction |  |  |
| Lifelong Interest in Global Health & Development |  |  |

**Additional file 1: Appendix 5: Descriptive Coding Layers**

**Benefits**

**Costs**

| **Costs for the Individual** | **Costs for the Institution** | **Costs for the UK** |
| --- | --- | --- |
| Exhaustion/Burnout/Stress | Loss of staff from other areas of work/ Challenges of organising cover | Negative perception of the UK where links are run badly |
| Neglect of relationships/Burden of Family or friends | Opportunity costs (e.g. CSR not being used for other benefits) | Loss of staff from other areas of work |
| Loss of annual leave | Management of security risks | Management of security risks |
| Accident/Injury | Trained staff leaving their post following links | Financial Cost |
| Culture shock | Negative perception of the UK institution where links are run badly |  |
| Imposing upon others when finding cover | Distracts staff from their work at the institution |  |
| Negative effects on career | Financial cost |  |
| Financial cost |  |  |
| Opportunity costs (e.g. missing conferences and meetings) |  |  |

| **Domain** | **Opportunities** | **Skills** | **Knowledge/ Understanding** | **Attitudes** |
| --- | --- | --- | --- | --- |
| **Clinical Skills** | Exposure to unusual pathologies | 1. Ability to work within other health systems 2. Clinical (without technology) | Knowledge of Tropical Diseases |  |
| **Management Skills** | Exposure to extra responsibilities | 1. Prioritisation of Limited Resources, 2. Problem-solving 3. Diplomacy 4. Self-understanding 5. Innovation 6. Ability to cope in different environments | Learning how to affect change | Appreciation of importance of openness to new ideas |
| **Communication and teamwork** | 1. To build productive relationships 2. To practice languages | 1. Team-working 2. Improved Languages |  | Appreciation of value of relationships |
| **Patient experience and dignity** |  |  | 1. Understanding of patients from different areas 2. Learning about other cultures |  |
| **Policy** | To utilise policy skills | Learning how to apply for grants | 1. Understanding of Other Health Systems 2. Learning how to affect change | Appreciation of value of NHS |
| **Academic skills** | 1. Opportunities to research unusual areas 2. Opportunities to undertake collaborative research 3. Opportunities to train/educate | 1. Learning how to apply for grants 2. Enhance skills in training delivery | Understanding of how to target training more effectively | Interest in researching new areas |
| **Personal satisfaction and interest** | Opportunities to train/educate |  | Understanding of needs of developing countries | 1. Perspective on UK problems 2. Appreciation of value of NHS 3. Life-long interest in global health |

**Additional file 1: Appendix 6 – Descriptive Coding of Individual Benefits**

**Additional file 1: Appendix 7 – Detailed Mapping of Individual Benefit Domains onto the Knowledge & Skills Framework’s most advanced descriptor (Level 4)**

| **Dimension** | **Level 4 descriptor** | **Related individual outcome measures** |
| --- | --- | --- |
| **Core Dimensions** | | |
| Communication | Develop and maintain communication with people on complex matters, issues and ideas and/or in complex situations | Teamwork, diplomacy, languages |
| Personal and people development | Develop oneself and others in areas of practice | Teamwork, education, leadership and management |
| Health, safety and security | Maintain and develop an environment and culture that improves health, safety and security | Leadership and management |
| Service improvement | Work in partnership with others to develop, take forward and evaluate direction, policies and strategies | Leadership and management, innovation, research and policy |
| Quality | Develop a culture that improves quality | Clinical skills, knowledge of tropical diseases |
| Equality and diversity | Develop a culture that promotes equality and values diversity | Culture, team work, global health understanding |
| **Health and Wellbeing Dimensions** | | |
| Promotion of health and wellbeing and prevention of adverse effects on health and wellbeing | Promote health and wellbeing and prevent adverse effects on health and wellbeing through contributing to the development, implementation and evaluation of related policies | Leadership and management, prioritisation of limited resources, research and policy |
| Assessment and care planning to meet health and wellbeing needs | Assess complex health and wellbeing needs and develop, monitor and review care plans to meet those needs | Clinical skills |
| Protection of health and wellbeing | Develop and lead on the implementation of an overall protection plan | Leadership and management, prioritisation of limited resources, clinical skills, research and policy |
| Enablement to address health and wellbeing needs | Empower people to realise and maintain their potential in relation to health and wellbeing | Cultural understanding, team-work |
| Provision of care to meet health and wellbeing needs | Plan, deliver and evaluate care to address  people’s complex health and wellbeing needs | Clinical skills, leadership and management |
| Assessment and treatment planning | Assess physiological and psychological functioning when there are complex and/or undifferentiated abnormalities, diseases and disorders and develop, monitor and review related treatment plans | Clinical skills, knowledge of tropical diseases |
| Interventions and treatments | Plan, deliver and evaluate interventions and/or treatments when there are complex issues and/or serious illness | Clinical skills, team work |
| Biomedical investigation and intervention | Plan, undertake, evaluate and report complex/unusual biomedical investigations and/or interventions | Clinical skills, knowledge of tropical diseases |
| Products to meet health and wellbeing needs | Support, monitor and control the supply of products | management, prioritisation of limited resources |
| **Estates and Facilities Dimensions** | | |
| Systems, vehicles and equipment | Review, develop and improve systems,  vehicles and equipment | Innovation, ability to work in other health systems |
| Environments and buildings | Plan, design and develop environments, buildings and/or items | Innovation |
| Transport and logistics | Plan, develop and evaluate the flow of people and/or items | Innovation, management |
| **Information and Knowledge** | | |
| Information processing | Develop and modify data and information management models and processes | Innovation |
| Information collection and analysis | Plan, develop and evaluate methods and processes for gathering, analysing, interpreting and presenting data and information | Management, research |
| Knowledge and information resources | Develop the acquisition, organisation, provision and use of knowledge and information | Education, research and policy |
| **General Dimensions** | | |
| Learning and development | Design, plan, implement and evaluate learning and development programmes | Education, research and policy |
| Development and innovation | Develop new and innovative concepts, models, methods, practices, products and equipment | Innovation, prioritisation of limited resources, leadership and management |
| Procurement and commissioning | Develop, review and improve commissioning and procurement systems | Teamwork, diplomacy, leadership and management |
| Financial management | Plan, implement, monitor and review the acquisition, allocation and management of financial resources | Teamwork, diplomacy, leadership and management |
| Services and project  management | Plan, coordinate and monitor the delivery of  services and/or projects | Leadership and management, prioritisation of limited resources, ability to work in other health systems |
| People management | Plan, develop, monitor and review the recruitment, deployment and management of people | Teamwork, leadership and management |
| Capacity and capability | Work in partnership with others to develop and sustain capacity and capability | Culture, teamwork, leadership and management |
| Public relations and  marketing | Plan, develop, monitor and review public relations and marketing for a service/organisation | Leadership and management |
